# Inhibition of the glucocorticoid receptor results in an enhanced miR-99a/100-mediated radiation response in stem-like cells from human prostate cancers

**DOI:** 10.18632/oncotarget.10207

**Published:** 2016-06-21

**Authors:** Jayant K. Rane, Holger H.H. Erb, Giovanna Nappo, Vincent M. Mann, Matthew S. Simms, Anne T. Collins, Tapio Visakorpi, Norman J. Maitland

**Affiliations:** ^1^ The Cancer Research Unit, Department of Biology, University of York, York, North Yorkshire, YO10 5DD, UK; ^2^ Hull York Medical School, University of Hull, Hull, East Yorkshire, HU6 7RX, UK; ^3^ Department of Urology, Castle Hill Hospital, Cottingham, East Yorkshire, HU16 5JQ, UK; ^4^ Prostate Cancer Research Center, Institute of Biosciences and Medical Technology - BioMediTech, University of Tampere and Tampere University Hospital, Tampere, 33520 Finland; ^5^ Leukaemia and Stem Cell Biology Group, Department of Haematological Medicine, King's College London, Rayne Institute, London, SE5 9NU, UK; ^6^ Laboratory of Molecular Haematopoiesis and Stem Cell Biology, Department of Experimental and Clinical Medicine, Magna Græcia University, 88100, Catanzaro, Italy

**Keywords:** cancer stem cells, miRNA, radiotherapy, DNA damage repair, mifepristone

## Abstract

Radiation therapy is a major primary treatment option for both localized early stage prostate cancer, and for advanced, regionally un-resectable, cancer. However, around 30% of patients still experience biochemical recurrence after radiation therapy within 10 years. Thus, identification of better biomarkers and new targets are urgently required to improve current therapeutic strategies. The miR-99 family has been shown to play an important role in the regulation of the DNA damage response, via targeting of the SWI/SNF chromatin remodeling factors, SMARCA5 and SMARCD1 in cell line models. In the present study, we have demonstrated that low expression of miR-99a and miR-100 is present in cell populations which are relatively radiation insensitive, for example in prostate cancer stem cells and in castration-resistant prostate cancer. Additionally, treatment of cells with the synthetic glucocorticoid, Dexamethasone resulted in decreased miR-99a and 100 expression, suggesting a new mechanism of miR-99a and 100 regulation in androgen-independent prostate cells. Strikingly, treatment of prostate cells with the glucocorticoid receptor inhibitor, Mifepristone was found to sensitize prostate cells to radiation by increasing the levels of miR-99a and miR-100. These results qualify the miR99 family as markers of radiation sensitivity and as potential therapeutic targets to improve efficiency of radiotherapy.

## INTRODUCTION

Radiation therapy (RT) is a major primary treatment option for localized early stage prostate cancer (PCa) and regionally un-resectable advanced PCa [1, 2]. Recently there have been significant improvements to RT methodology, resulting in an increase of recurrence-free survival [3]. However, around 30% of patients still experience biochemical recurrence after RT within 10 years, for which there is no consensus regarding optimal management [4]. One of the main causes of the varied response to RT is the high inter- and intra-tumoral heterogeneity found in PCa [5, 6]. Moreover, this heterogeneity is primarily responsible for the current lack of markers to group patients into high- and low-risk for relapse, which consequently results in over-treatment of 20–42% of patients [7].

Several studies have demonstrated that a small population of primitive stem-like cells (cancer stem cells; CSC) within the tumor are more resistant to radiotherapy than the majority of cells, and are directly responsible for tumor recurrence [8, 9]. In PCa, cell populations with the CD44^+^/α_2_β_1_integrin^hi^/CD133^+^, CD49f^hi^/Trop2^hi^, and CD44^+^/CD49f^hi^/Trop2^+^ phenotype have been shown to share CSC properties [10–14]. However, these markers have not been used to stratify patients on the basis of their radiosensitivity.

MicroRNAs (miRNAs) have demonstrable potential as diagnostic, predictive, and prognostic markers, and may provide a promising new class of therapeutic targets [15–17]. MiRNAs are small 17–25 nucleotide non-coding RNA molecules, which regulate post-transcriptional gene expression in a sequence-specific manner and have a central role in multiple biological functions, including cell survival, proliferation, and DNA damage responses [18–20]. Several miRNAs can share a nearly identical seed sequence and are likely to target the same sets of mRNAs. These miRNAs have been grouped together in “miRNA families”. The miR-99 family (miR-99a, miR-99b, and miR-100) has been reported to be upregulated following DNA damage, and their expression has been correlated with radiation sensitivity, in breast and PCa cell lines, by their ability to downregulate the chromatin remodeler SWI/SNF-related, matrix-associated, actin-dependent regulator of chromatin (SMARC) A5 (SNF2H) [21]. Thus, induction of the members of the miR-99 family represents a switch by which cells subjected to multiple rounds of radiation might be sensitized to RT. The precise molecular mechanism by which RT induces cell death has not been defined, however a failure to repair DNA damage seems to be one of the main causes [22]. Although RT is a predominant front-line treatment, it is also known to cause several side effects (including pain, fatigue and sexual, urinary and bowel dysfunction) which have a detrimental effect on quality of life [23]. Therefore, in order to effectively manage RT-side effects, a lower dose of radiation, optimized to achieve the same results, would be an ideal therapeutic strategy.

This study shows, for the first time the role of two members of the miR-99 family (miR-99a and miR-100) in DNA damage repair following radiation in primary PCa cell models, and provides additional functional and mechanistic details about the miR-99a family-DNA repair relationship. These miRNAs are expressed at only low levels in the stem-like RT-resistant CD44^+^/α_2_β_1_integrin^hi^/CD133^+^ subpopulations from benign and cancerous prostate tissue, supporting their role in treatment resistance and cancer relapse [8]. In addition, we show that miR-99a and miR-100-mediated radiation-sensitivity is influenced by inhibition of the Glucocorticoid receptor (GR, NRC1), revealing a potential new treatment strategy to improve radiotherapy and reduce PCa relapse.

## RESULTS

### Lower expression of miR-99a and miR-100 is associated with aggressive PCa and a stem cell-like phenotype

**Figure 1:**
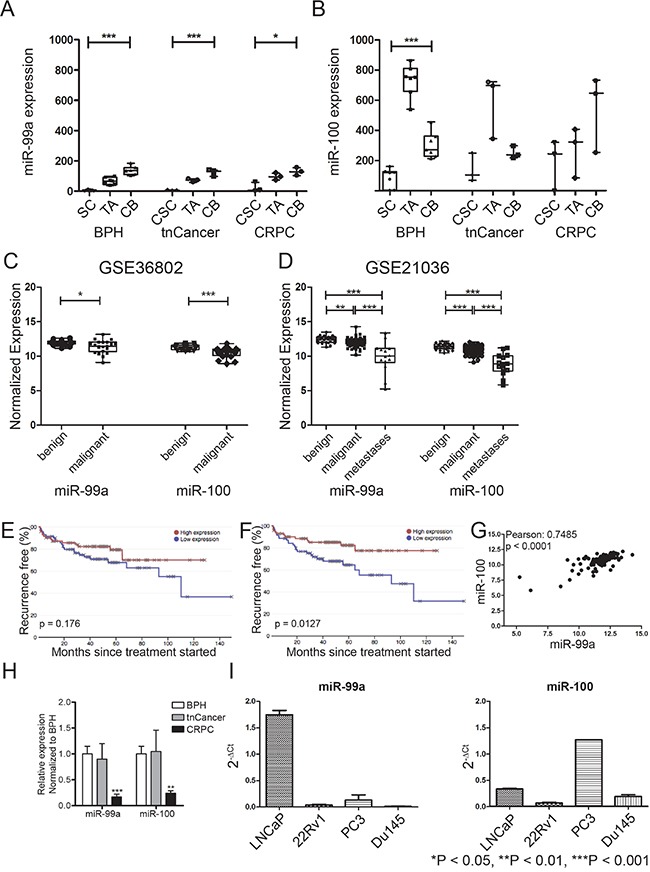
miR-99a/100 function together and their lower expression imparts aggressive PCa disease and stem cell-like phenotype **A**+**B.** Expression profiles of miR-99a (A) and miR-100 (B) in the separated populations: stem cell (SC), cancer stem cell (CSC), transit amplifying (TA) and committed basal (CB) (n=5 Benign prostatic hyperplasia (BPH) and treatment naïve Prostate Cancer (tnCancer), n=3 castration resistant PCa (CRPC). **C**+**D.** miR-99a and miR-100 levels in unseparated benign and malignant populations from the GSE21036 (C, benign n=28, malignant n=99, metastasis n=14) and GSE36802 (D, benign n=21, malignant n=21) data sets. **E**+**F.** Survival analysis from GSE21036 of patients with low and high mir-99a (E) and miR-100 (F) levels using the Project Betastasis database (http://www.betastasis.com/prostate_cancer/taylor_et_al_2010/kaplan-meier_survival_plot/28/02/2016). The median was chosen as threshold. **G.** Correlation analysis of miR-99a and miR-100 after pooling the expression data of GSE21036 and GSE36802. The analysis shows a significant correlation between miR-99a and miR-100 expression in PCa patients. **H.** Comparison of miR-99 and miR-100 expression in unfractionated primary prostate samples from BPH (n=3), tnCancer (n=3) and CRPC (n=3). **I.** Expression profiles of miR-99 and miR-100 in prostate cancer cell lines (n=3). Data are expressed as mean ± s.d. *P < 0.05, **P < 0.01, ***P < 0.001 (Student's ttest).

Analysis of our published miRNA expression array data demonstrates that the miR-99 family members, miR-99a and miR-100 (miR-99a/100), are significantly suppressed in prostate stem-like cells (SC) compared to their differentiated progeny; committed basal (CB) cells (Figure 1A, 1B) [24, 25]. This was true for SCs and CBs enriched from benign prostatic hyperplasia (BPH), treatment naïve PCa (tnCancer), and castration-resistant PCa (CRPC) samples. Analysis of two further expression arrays published by other groups revealed that miR-99a and miR-100 are also significantly suppressed in primary tumors compared to benign samples (Figure 1C, 1D) [26, 27]. Interestingly, data from Taylor et al (2012) showed that the expression of these two miRNAs is further suppressed in metastatic PCa samples compared to treatment naïve cancers (P<0.001) (Figure 1D). These data are consistent with other large-scale sequencing studies, which have also reported a decrease of the miR-99 family in PCa [28–30]. Although levels exhibit no correlation with Gleason grade (Supplementary Figures S1A, S1B), Kaplan- Meier survival analysis on the Taylor et al data showed that lower expression of miR-99a/100 is associated with poorer survival (Figure 1E, 1F). Additionally, miR-99a/100 were also found to be significantly co-expressed in prostate samples (Pearson: 0.07485, p<0.0001) (Figure 1G). In support of these findings we observed that in patient-derived epithelial cells, miR-99a/100 expression was significantly suppressed in CRPC compared to benign disease and tnCancer (P<0.01) (Figure 1H). Quantitative real time polymerase chain reaction (qRT-PCR) analysis of commonly used cell lines showed that more tumorigenic PCa cell lines, such as DU145 and 22Rv1, had a lower expression of the miR-99 family than less tumorigenic PCa cell lines, e.g. LNCaP (Figure 1I). Taken together, these data suggest that miR-99a/100 function together, and that their lower expression imparts aggressive PCa disease and a stem cell-like phenotype in a variety of human PCa models.

### Suppression of miR-99a and miR-100 promotes efficient DNA repair in cells with high miR-99a/100 expression

**Figure 2:**
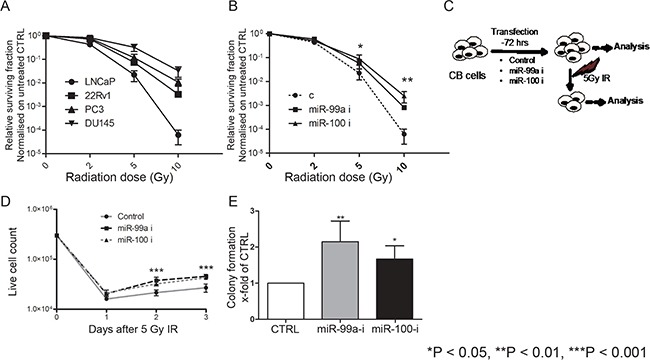
Suppression of miR-99a and miR-100 promotes efficient DNA repair in cells with high miR-99a/100 expression **A.** Proliferation assays showing the relative surviving fraction of PCa cell lines 48 h after exposing them to 2, 5, and 10 Gy radiation (n=3). **B.** Proliferation assays showing the relative surviving fraction of LNCaP cells transfected with control, miR-99a-inhibitor, and miR-100-inhibitor cells 48 h after exposing the to 2, 5, 10 Gy radiation (n=3). **C.** Schematic representation of methodology for miRNA inhibition experiments. Committed basal (CB) cells, which express relatively high levels of miR-99a/100, were transfected with control, miR-99a-inihibitor or miR-100 inhibitor for 3 days and then analyzed with or without exposure to 5-Gy radiation. **D.** Proliferation of malignant irradiated CB cells measured by live cell count after miR-99a and miR-100 inhibition (n=3 PCa). **E.** Colony forming assay of malignant irradiated CB cells after miR-99a and miR-100 inhibition (n=3 PCa). Data are expressed as mean ± s.d. *P < 0.05, **P < 0.01, ***P < 0.001 (Student's ttest).

A previous study showed that higher expression of the miR-99 family correlated with radiation sensitivity of prostate cell lines [21]. Cell viability assays revealed that the radiation sensitivity of the tested PCa cell lines (Figure 2A) is higher in cells with low expression of the miR-99 family (Figure 1I). Furthermore, inhibition of miR-99a/100 in LNCaP cells (Supplementary Figure S1D) increases radioresistance (Figure 2B). To further investigate the role of miR-99a/100 in radiation response, we inhibited miR-99a or miR-100 expression in highly expressing BPH and PCa-derived primary CB cells (Figure 2C). Inhibition of miR-99a/100 resulted in a growth advantage (Figure 2D), and we observed a significant (~2-fold) increase in colony forming efficiency following exposure to 5 Gy radiation (Figure 2D, 2E).

### Suppression of miR-99a/100 promotes recruitment of DNA repair proteins

**Figure 3:**
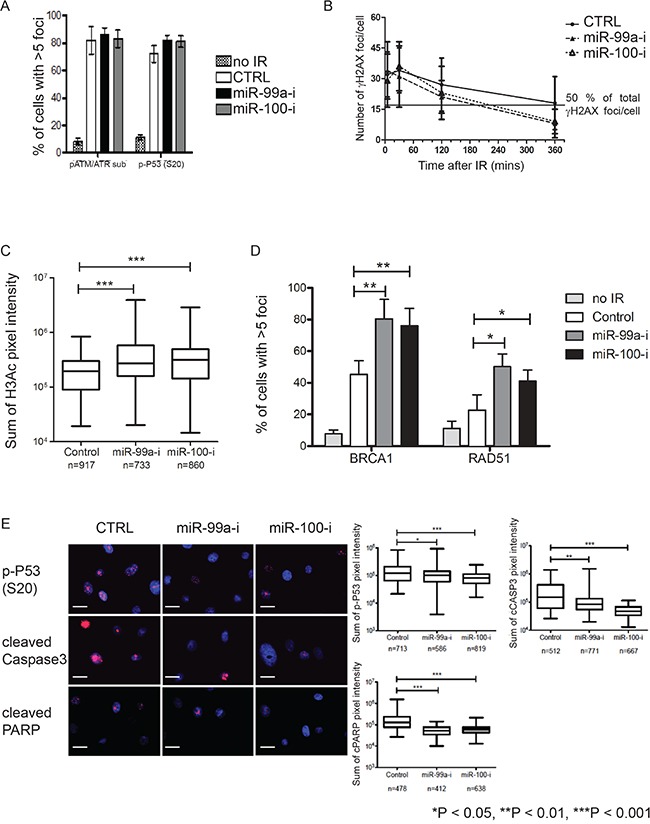
Suppression of miR-99a and 100 promotes DNA repair enhance recruitment of DNA repair proteins **A.** Quantification of positive nuclear phospho-ATM/ATR substrate and phospho-P53 (s-20) stained CB cells transfected with miR-99a and 100 inhibitor. Immunofluorescence staining was performed was performed 30 minutes after exposure to 5-Gy (n=3 PCa, each sample in triplicate), >250 cells/sample were counted. **B.** Quantification of γH2AX immunofluorescence foci/nucleus at multiple time points after transfection of miR-99a/100 inhibitor in CB cells following 5-Gy radiation exposure (n=3 PCa, each sample in triplicate), >250 cells/sample were counted. Line represents 50% of total γH2AX foci/cell. **C.** Quantification of nuclear pan-histone 3-acetylation immunofluorescence staining intensity by Velocity Quantitation software in miR-99a and 100-inhibitor transfected CB cells 30 minutes after exposure to 5-Gy radiation (n=3 PCa). n indicates total number of cells included in the analysis. **D.** Quantification of miR-inhibitor transfected CB cells exhibiting nuclear BRCA1 and RAD51 immunofluorescence foci, 120 minutes after exposure to 5-Gy radiation (n = 3 PCa, each sample in triplicate), >250 cells/sample were counted. **E.** Representative pictures of immunofluorescence staining for phosphop53 (s-20), cleaved caspase 3, and cleaved PARP expression in miR-99a/100 inhibitor transfected CB cells, 24 h after exposure to 5-Gy radiation (n=3 PCa, each sample in triplicate). The right panel shows quantitation of staining using Velocity Quantitation software. n indicates total number of cells quantified. Scale bar: 120 μm. Data are expressed as mean ± s.d. *P < 0.05, **P < 0.01, ***P < 0.001 (Student's ttest).

Since our results showed that inhibition of miR-99a/100 expression led to a faster recovery of CB cells after irradiation (Figure 2D, 2E), we next quantified levels of DNA damage in CB cells with or without miR-99a/100 inhibition. Nuclear pATM/ATR substrate and phosphorylated p53 levels were measured 15 min after exposure to 5 Gy radiation (Figure 3A), but the number of cells with >5 foci did not change. To elucidate the mechanism(s) behind the role of miR-99a/100 in DNA repair, we investigated the potential roles of chromatin remodeling and DNA repair proteins. Increased phosphorylation of histone H2AX at serine 139 (γH2AX) has previously been reported as the most sensitive marker of DNA damage, where decreased phosphorylation reflects subsequent repair of the DNA lesion [31]. To monitor damage and repair of the DNA, the number of γH2AX foci per cell, after irradiation of miR-99a/100 inhibited CB cells were estimated by immunofluorescence. Under all conditions γH2AX peaked at the same level in the first 30 min post-irradiation, but 215 min after irradiation, the cells transfected with miR99a/100 inhibitors showed a 50% decrease in the number of γH2AX foci. Scrambled controls failed to achieve this 50% decrease until 360 min post-irradiation (Figure 3B). This finding, combined with the earlier observation that these cells recover faster after irradiation, led us to formulate the hypothesis that DNA damage is repaired more rapidly after miR-99a/100 inhibition. Assessment of the total pixel intensity of the nuclear chromatin accessibility marker, Histone 3 acetylation (H3ac), after 30 min, showed an increased histone H3ac after miR-99a/100 inhibition (Figure 3C). Using the same technique, we observed a significant increase in the DNA damage sensors BRCA1 and RAD51 in miR-99a/100- inhibited cells 2-hours after exposure to 5Gy irradiation (Figure 3D). In support of our findings, phosphorylation of the damage sensor protein p53, and of the apoptotic markers cleaved caspase 3 and cleaved PARP, both showed a significant decrease in cells exposed to miR-99a/100 inhibition 24 h after irradiation (Figure 3E). These data provide further evidence that lower expression of miR-99a/100 permits efficient DNA repair, whilst expression of miR-99a/100 induces p53-dependent apoptosis following DNA damage.

### miR-99a/100 inhibition-dependent DNA repair is mediated by SMARCA5 and SMARCD1

**Figure 4:**
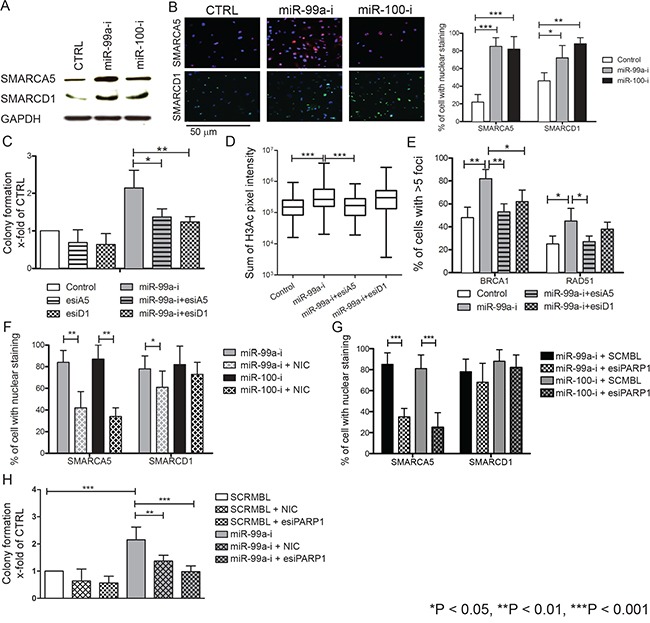
Effects of miR-99a and 100 on DNA repair processes are regulated by SMARCA5 and SMARCD1 **A.** Representative western blot analysis of SMARCA5 and SMARCD1 expression in miR-inhibitor transfected malignant CB cells. **B.** Immunofluorescence staining for nuclear SMARCA5 and SMARCD1 in miR-99a and 100-inhibitor transfected CB cells, 5 minutes after exposure to 5-Gy radiation (n=5 PCa). Scale bar: 100 uM. Right panel shows the quantification of SMARCA5 and SMARCD1 positive CB cells, >250 cells/sample counted and SMARCA5 and SMARCD1 fluorescence quantification using Velocity quantitation software. **C.** Colony forming experiments of CB cells transfected simultaneously with miR-99a inhibitor and SMARCA5 or SMARCD1 endoribonuclease-prepared siRNA (esiRNAs) following 5-Gy radiation show a rescue of the effects mediated by miR-99a inhibitor alone (n=5 PCa, each sample is in triplicate). **D.** Quantification of nuclear pan-histone 3-acetylation immunofluorescence staining by Velocity Quantitation software in miR-99a inhibitor and SMARCA5 or SMARCD1 esiRNAs transfected CB cells. Immunofluorescence staining was performed 30 minutes after exposure to 5-Gy radiation (n=3 PCa, each sample in triplicate). n indicates total number of cells included in quantification analysis. **E.** Quantification of nuclear BRCA1 and RAD51 in CB cells simultaneously transfected miR-99a inhibitor and SMARCA5 or SMARCD1 esiRNA. Immunofluorescence staining was performed 120 minutes after exposure to 5-Gy radiation (n=3 PCa, each sample in triplicate), >250 cells/sample were counted. **F.** Quantification of nuclear SMARCA5 and SMARCD1 in CB cells simultaneously transfected with miR-99a inhibitor and treated with or without the PARP1 inhibitor nicotinamide. Immunofluorescence staining was performed 120 minutes after exposure to 5-Gy radiation (n=5 PCa, each sample in triplicate), >250 cells/sample were counted. **G.** Quantification of nuclear SMARCA5 and SMARCD1 in CB cells simultaneously transfected with miR-99a inhibitor and PARP1 esiRNA. Immunofluorescence staining was performed 120 minutes after exposure to 5-Gy radiation (n=5 PCa, each sample in triplicate), >250 cells/sample were counted. **H.** Colony forming experiments of CB cells transfected with SCRMBL or PARP1 esiRNA or treated with nicotinamide following by 5-Gy radiation showing a rescue of the effects mediated by miR-99a inhibitor alone (n=5 PCa, each sample in triplicate). Data are expressed as mean ± s.d. *P < 0.05, **P < 0.01, ***P < 0.001 (Student's ttest).

SMARCA5 and SMARCD1 are major components of the SWI/SNF chromatin remodeling complex, both of which play essential roles in DNA damage repair and cell survival post-DNA damage [32]. Luciferase 3′UTR-studies using PCa cell lines have shown that SMARCA5 can be regulated by miR-99a/100 and influences proliferation, PSA protein levels and repair of double-strand DNA breaks [21, 30]. Using primary PCa cells, we investigated this relationship in detail to obtain more mechanistic data in the context of cancer stem cells. As reported in other systems, SMARCA5 and SMARCD1 proteins were upregulated (Figure 4A) after inhibition of miR-99a/100 [21, 30]. Since SMARCA5 is known to be rapidly recruited at DNA damage sites in the nucleus [33], we measured post-radiation nuclear SMARCA5 and SMARCD1 accumulation in CB cells, using immunofluorescence. Both proteins reached their highest nuclear levels after 3 minutes and began to decline after 5 min (Supplementary Figure S2A). Accordingly, nuclear SMARCA5 and SMARCD1 levels were measured in miR-99a/100 inhibited CB populations 5 min after irradiation. miR-99a/100 inhibited CB cells showed significantly higher nuclear SMARCA5 and SMARCD1 accumulation compared with scrambled miRNA transfected cells (Figure 4B). Similarly, the low miR-99a/100 expressing SC fraction accumulated significantly higher levels of the SMARCA5 and SMARCD1 in the nucleus, compared with the high miR-99a/100 expressing CB population after radiation exposure (Supplementary Figure S2B). Therefore, miR-99a/100 influences DNA repair via regulation of SMARCA5 and SMARCD1, even in primary PCa cells. To further validate this, we attempted to reverse the efficient DNA repair ability acquired by CB cells, due to inhibition of miR-99a, by simultaneously knocking down expression of SMARCA5 or SMARCD1. Simultaneous inhibition of SMARA5/SMARCD1 and miR-99a reduced the post-radiation colony recovery ability of CB cells (Figure 4C). CB cells transfected with miR-99a inhibitor showed an increase in chromatin relaxation by increasing H3-acetylation (Figure 4D), resulting in efficient nuclear recruitment of BRCA1 and RAD51 (Figure 3D, 4E). Simultaneous inhibition of SMARCA5 and miR99-a, but not of SMARCD1, reduced H3-acetylation suggesting that miR-99a mediated chromatin relaxation is predominantly mediated by SMARCA5 only. Similarly, concurrent miR-99a inhibition and SMARCA5/SMARCD1 knock-down abrogated efficient BRCA1 and RAD51 nuclear recruitment at the DNA damage sites (Figure 4E). These molecular and functional readouts revealed that miR-99a/100 regulate SMARCA5 and SMARCD1 in primary PCa cells to enable DNA repair.

### SMARCA5 and SMARCD1 mediated DNA repair is dependent on PARP1

Previous studies have shown that poly ADP ribose polymerase (PARP)1 is essential for SMARCA5 recruitment at double-strand DNA break sites in the human osteosarcoma cell line U-2 OS [34]. We have previously shown that CSCs are more radioresistant than CB cells and PARP1 is specifically overexpressed in CSCs (Supplement Figure S2C) [8, 35]. Therefore, we further hypothesized that PARP proteins play an essential role in recruitment of SMARCA5 and SMARCD1 at DNA break sites in primary prostate cells. To inhibit PARP activity, the non-specific PARP activity inhibitor, nicotinamide and PARP1 endoribonuclease-prepared siRNA (esiRNA) were used [36]. When CB cells, where miR-99a expression was inhibited, were treated with 15 μM nicotinamide, for 12 hours, and then irradiated with 5Gy radiation, we observed that the post-radiation nuclear accumulation of SMARCA5/SMARCD1 was significantly reduced (Figure 4F). A similar reduction in SMARCA5/SMARCD1 post-radiation nuclear localization was also observed when CB cells were co-transfected with miR-99a inhibitors and esiPARP1 (Figure 4G). PARP1 inhibition ultimately negated the post-radiation survival advantage imparted by miR-99a inhibition in CB cells (Figure 4H), suggesting that PARP1 is required for post-radiation nuclear accumulation of SMARCA5.

### Suppression of miR-99a/100-induced efficient DNA repair in CB cells is not due to epithelial–mesenchymal transition or de-differentiation

**Figure 5:**
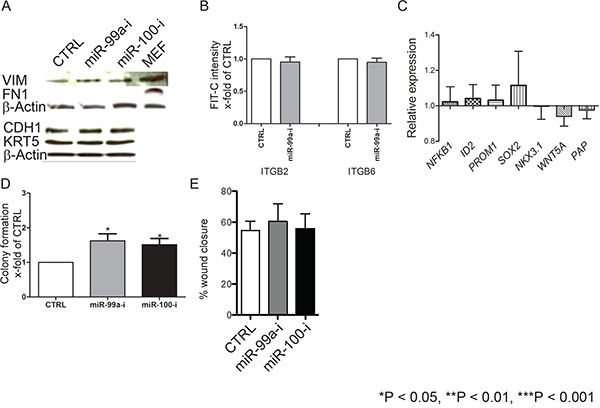
Suppression of miR-99a/100-induced efficient DNA repair in CB cells is not due to induction of epithelial–mesenchymal transition or de-differentiation **A.** Representative western blot analysis of epithelial-mesenchymal transition-associated proteins E-cadherin (CDH1), fibronectin (FN1) and Vimentin (VIM) in CB cells transfected with control, miR-99a-inhibitor, and miR-100-inhibitor, for 3 days. **B.** FACS analysis for CD49b (ITGB2) and CD49f (ITGB6) expression of CB cells transfected with either control or miR-99a inhibitor for 3 days (n=3 PCa). **C.** mRNA levels of differentiation-associated genes (Nuclear factor kappa-light-chain-enhancer of activated B cells 1 (NFkB1), DNA-binding protein inhibitor ID-2 (ID2), prominin 1 (PROM1), Sex determining region Y-box 2 (SOX2), Homeobox protein Nkx-3.1 (NKX3.1), Wingless-Type MMTV Integration Site Family, Member 5A (WNT5a) and Pappalysin A (PAP)) after miR-99a-inhibitor transfection in CB cells, for 3 days, relative to control transfection. None of the changes were statistically significant (n = 2 BPH and 3 PCa, each sample in triplicate) were measured by qRT-PCR and normalized to RPLP0. **D.** Colony forming efficiency of miR-99a/100 inhibitor transfected CB cells (n=3 PCa). **E.** Wound healing assay miR-99a/100 inhibitor transfected CB cells after 48 h. Data are expressed as mean ± s.d. *P < 0.05, **P < 0.01, ***P < 0.001 (Student's ttest).

It is well established that cells undergoing epithelial-mesenchymal transition (EMT) and SCs are more radioresistant [37, 38]. Lower expression of miR-99a/100 and higher expression of SMARCA5/SMARCD1, and even PARP1 have all been associated with EMT and the stem cell phenotype in various other tissue types [39–45]. We therefore investigated whether induction of DNA repair via the miR-99a/100-SMARA5/SMARCD1 axis was due to either EMT or de-differentiation of CB cells into SC. We inhibited miR-99a/100 in CB cells and looked for EMT or dedifferentiation, using commonly used EMT and previously reported PCa and normal stem cell markers [10, 11, 35, 46–48]. None of these markers showed any changes after inhibition of miR-99a/100 (Figure 5A, 5B, 5C). Colony forming efficiency is often used as a surrogate to functionally assess stem-ness. Inhibition of miR-99a/100 inhibition produced only a modest, but significant, increase in colony forming efficiency of CB cells (Figure 5D). Moreover a scratch assay demonstrated that miR-99a/100 inhibited CB cells did not increase their migratory potential (Figure 5E), an attribute of mesenchymal-like cells. These data provide multiple strands of evidence that miR-99a/100 inhibition in CB cells did not undergo EMT not de-differentiation, as a basis for radiation resistance.

### Glucocorticoids downregulate miR-99a/100 expression levels

**Figure 6:**
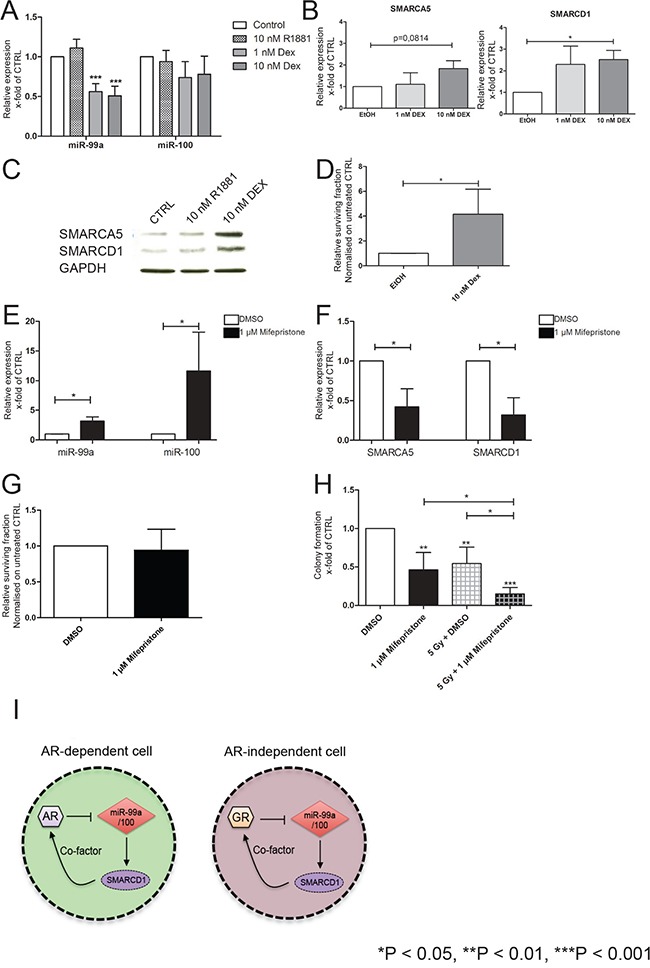
Effects of miR-99a/100 on DNA repair processes are regulated by SMARCA5 and SMARCD1 **A.** qRT-PCR analysis of miR-99a and miR-100 expression in CB cells treated with R1881 or dexamethasone (DEX) for 72 h (n= 5 PCa). **B.** qRT-PCR analysis of SMARCA5 and SMARCD1 expression in CB cells treated with DEX for 72 h (n= 5 PCa). **C.** Representative western blot analysis of SMARCA5 and SMARCD1 in CB cells treated with R1881 or DEX for 72 h. **D.** Cell viability assay after 72 h of CB cells exposed to DEX for 72 h followed by irradiation (Gy 5, n=3 PCa). **E.** qRT-PCR analysis of miR-99a and miR-100 expression in total primary cell populations treated with Mifepristone for 72 h (n= 5 PCa). **F.** qRT-PCR analysis of SMARCA5 and SMARCD1 expression in total primary cell populations treated with Mifepristone for 72 h (n= 5 PCa). **G.** Cell viability assay after 72 h of total primary cell populations after exposure to DEX for 72 h followed by irradiation (Gy 5, n=5 PCa). **H.** Colony forming efficiency of primary prostate cells after being exposure to Mifepristone for 72 h followed by irradiation (Gy 5, n=5 PCa). **I.** Schematic representation of the hypothesis, which proposes a feedback loop between androgen receptor (AR)-miR99a/100-SMARCD1 and glucocorticoid receptor (GR)-miR99a/100-SMARCD1 in androgen dependent and androgen independent cells. Data are expressed as mean ± s.d. *P < 0.05, **P < 0.01, ***P < 0.001 (Student's ttest).

We have previously shown that androgen regulated genes in luminal cells can also be controlled by a different steroid hormone in androgen-independent basal cells [48]. A previous study suggested that miR-99a/100 are suppressed by androgens in androgen-dependent cells with a luminal phenotype [30]. However it is known that miR-100 expression in human (androgen independent) corneal fibroblasts is significantly suppressed by synthetic glucocorticoid Dexamethasone (DEX) [49]. DEX treatment also induces resistance to radiation and cytotoxic therapy in multiple (androgen independent) human cancer types [50–52]. Our previous data showed that glucocorticoid receptor (GR/NR3C1) expression is higher in primary prostate normal and cancer stem cells, compared to CB cells from normal and cancer primary cultures (Supplement Figure S2D). Therefore, when CB populations were treated with DEX the expected lower expression of both miR-99a/miR-100, and a reciprocally increased expression of SMARCA5 and SMARCD1 mRNA, compared with ethanol (EtOH) treated cells (Figure 6A, 6B) was observed. Treatment with 10 nM DEX also increased SMARCA5 and SMARCD1 protein levels in CB cells 72 h after treatment (Figure 6C). However, treatment of androgen-independent CB and PC3 cells, with the synthetic androgen R1881 (10 nM), did not result in a change of miR-99a/miR-100 or SMARCA5 and SMARCD1 expression (Figure 6A, 6B, Supplementary Figure S3A), whereas LNCaP, an AR expressing PCa cell line demonstrated a downregulation of both miRNAs after R1881 treatment (Supplementary Figure S3B), confirming previous data [30]. Whilst in androgen-dependent LNCaP cells, treatment with the anti-androgen Bicalutamide (BC) reverses the down-regulation of miR-99a/100, no BC effects were seen in androgen-independent PC3 cells (Supplementary Figure S3A, S3B). Subsequently, when we measured the viability of DEX-treated near-patient CB cells after irradiation, the DEX-treated population contained 4 fold more viable cells after irradiation compared to the control population (Figure 6D). Our results show that inhibition of miR-99a/miR-100 via glucocorticoid treatment results in an increased DNA repair efficiency at least partly through regulation of the SMARCA5 and SMARCD1 proteins in androgen-independent cells.

### Inhibition of the glucocorticoid receptor upregulates miR-99a/100 expression levels

Having demonstrated that stimulation of the GR with DEX led to suppression of miR-99a/100 expression (Figure 6A, 6B), total cell populations of patient-derived prostate cells were treated with the GR antagonist Mifepristone at the clinically achievable concentration of 1 μM [53]. miR-99a/miR-100 were significantly upregulated in the treated samples (Figure 6E). qRT-PCR analysis of the miR-99a and miR-100 targets SMARCA5 and SMARCD1 showed the expected decrease of both targets after Mifepristone treatment (Figure 6F). When Mifepristone treated cells were irradiated (5 Gy), cell viability showed no changes between Mifepristone and Dimethyl sulfoxide (DMSO) pre-treated cells (Figure 6F), but a significant decrease in clonogenic potential was observed with mifepristone treatment, which was further reduced after irradiation (Figure 6G). These data revealed that miR-99a/100 are regulated by glucocorticoids and influence DNA repair efficiency by modulating SMARCA5 and SMARCD1 in androgen-independent primary PCa cells (Figure 6I), with particular activity within the highly clonogenic stem-like cells.

## DISCUSSION

Recent studies have demonstrated that cells possessing a basal phenotype in the human prostate play an important role in tumor relapse and development of aggressive cancer [9, 54]. These cells represent less than 1% of the overall tumor mass and are highly resistant to commonly used therapies in PCa [9]. miRNAs have been shown to play a key role in chemotherapeutic drug resistance, and we now show that miR-99a/miR-100 are downregulated in patients with CRPC compared with benign disease. Interestingly, the SC and CSC miRNA signatures were recapitulated in unfractionated CRPC samples, but not in treatment-naïve cancers. This is in agreement with other studies, which have shown that SC and CSC mRNA and miRNA signatures are similar to those of unfractionated CRPC [54–56]. Furthermore, patients with low levels of miR-99a/miR-100 are more susceptible to biochemical recurrence after treatment. Taken together, these data reveal the potential of the miR-99 family as a marker for bad prognosis.

In our previous work we integrated miRNA-mRNA expression datasets and demonstrated that miR-99a/miR-100 plays an essential role in DNA repair [25, 35]. In this study, using patient-derived cells, we have shown that inhibition of miR-99a/miR-100 prevents p53 dependent apoptosis in PCa cells after irradiation. Moreover, suppressed miR-99a/100 levels enable efficient relaxation of damaged chromatin by increasing histone acetylation and subsequently increasing the recruitment of DNA repair proteins, BRCA1 and RAD51. Using loss of function and rescue experiments, we now demonstrate that SMARCA5 and SMARD1 are the primary mediators of the miR-99a/100 driven pathway. This result agrees with previous findings by Mueller et al, who showed a role for the miR-99 family in DNA repair [21].

We also noted that inhibition of miR-99a/100 (and overexpression of SMARCA5 and SMARCD1) resulted in small but significant increase in colony forming efficiency, but other stem cell markers remain statistically unchanged. Perhaps miR-99a/100 inhibition or expression of SMARCA5/SMARCD1 alone is required but not sufficient for de-differentiation. Previous data showed that concomitant overexpression of proteins such as EZH2 along with SMARCA5 overexpression is needed for epithelial stem cell maintenance [57]. EZH2 has also been shown to be a critical regulator of stem cell functionality, radio-resistance, and prostate cancer aggressiveness [58–60]. It is indeed possible that EZH2 and miR-99a/100 can collaborate in regulating prostate cancer stem cell functionality and radiation-sensitivity.

Our results also show that PARP1, whose expression is essential for the miR-99a/100 driven DNA damage response, is an important component of this process. PARP1 is essential for the maintenance of genomic integrity, due to its roles in DNA repair, chromatin remodeling, and transcription factor regulation [61]. PARP inhibition has recently been shown, in a subset of PCa patients, to expand survival times and increase radiosensitivity in xenografts [62, 63]. Based on these findings, PARP inhibition seems plausible as a potential enhancer of radiation therapy in PCa. Our results show that in cells treated with esiRNA against PARP1 or with the non-selective PARP inhibitor nicotinamide, recruitment of the DNA repair protein SMARCA5 and, to a smaller extent, SMARCD1 is inhibited. Inhibition of PARP ultimately interferes with the miR-99a/100-SMARCA5/SMARCD1 axis and thus DNA repair. These results provide evidence of an important mechanism by which PARP1 inhibition can result in radiosensitization in human cancers.

However, studies in prostate and other cancer types, have also reported PARP inhibitor resistance as a result of EMT, which is often present in cancers that acquire resistance to treatment [64–66]. In general, EMT is described as reprogramming of terminally differentiated cells into more mesenchymal-type cells [67]. Since we saw no changes in the expression of EMT markers after miR-99a/100 inhibition, the mechanism behind the change in DNA damage response after inhibition of miR-99a or miR100 is not due to dedifferentiation/EMT.

Our data demonstrate that the expression of miR-99a/100 is also regulated by GR control of the DNA damage response following irradiation. The exact mechanism by which GR influences miRNAs dosage has not yet been clarified, however it was previously shown that glucocorticoids could influence miRNA-processing enzymes [68]. Since miRNA are encoded in non-protein-coding regions and often intronic elements, there is also the possibility that regulation can be influenced by a GR response element, as has been reported for miR-708 [62]. miR-99a has been found in the lncRNA host gene MIR99AHG. Although MIR99AHG was shown to play an important role in leukaemia, little is known about its regulation [69]. miR-100 is located in an intronic area of the BH3-like motif-containing cell death inducer BLID, (https://omim.org/entry/615965) which has not been reported to be regulated by the GR and according to the Transcriptional Regulatory Element Database the gene has no cis- or trans- GR response elements (https://cb.utdallas.edu/cgi-bin/TRED/tred.cgi?process=home). These data therefore suggest that there could be an indirect influence of GR on these miRNAs. Glucocorticoids are widely used with adjuvant chemotherapy to attenuate off target toxicity and nausea. However, usage of glucocorticoids remains controversial; On the one hand, reduction of negative side effects during therapy using glucocorticoids is quite successful, but there are increasing reports suggesting that glucocorticoids can counteract taxane-based therapies, induce therapy resistance and support growth of aggressive tumor phenotypes [70–73]. A recent study of GR inhibition demonstrated reversion of docetaxel resistance in PCa [74]. Docetaxel resistant cancer and metastatic CRPC share several features with CSCs [72, 75, 76]. Our results show high expression of the GR in the CSC population, which has been shown to be highly chemo- and radio-therapy resistant, and therefore thought to play a role in tumor relapse [8, 9]. Here we show that pre-treatment with Dexamethasone or the GR inhibitor Mifepristone resulted in sensitivity changes of primary PCa to irradiation, by directly influencing the expression levels of miR-99a/100. These data not only implicate GR in resistance to RT in PCa cells, but also highlight the role of GR and miR99a/100 in the development of RT resistant tumors. However, due to the limited knowledge of interaction and regulation of noncoding RNAs and how they together contribute to disease, there remain many challenges before miRNA-based therapies can be realized in treatment approaches. In contrast, the indirect regulation of miRNA via already established and FDA-approved therapies seems to be more promising. Since GR inhibitors in CRPC patients are well tolerated, the use of GR inhibitors would be an acceptable means of enhancing RT efficiency and may potentially be a way to reduce tumor relapse frequencies [77].

We have shown in multiple near-patient PCa samples that the two miR-99 family members miR-99a/miR-100 play an important role in regulation of post-irradiation DNA damage response (via SMARC proteins) in the rare tumor initiating CSC population. The miRNAs can be upregulated by inhibition of the glucocorticoid receptor prior to radiotherapy. Therefore a combination therapy of GR inhibitors with RT could potentially enhance the efficiency of RT in PCa.

## MATERIALS AND METHODS

### Culture of cell lines and primary prostate cells

The PCa cell lines 22Rv1, LNCaP, PC3, and Du-145 were obtained from ATCC (Rockville, MD, USA) and where cultured as previously described [78]. Benign and cancerous primary prostate cells were cultured as described earlier [8, 10, 79]. Primary prostate cells were further fractionated into stem cell populations (SC, CD44^+^/α_2_β_1_integrin^hi^/CD133^+^), transit amplifying (TA) populations (CD44^+^/α_2_β_1_integrin^hi^/CD133^−^, TA), and committed basal populations (CB, CD44^+^/α_2_β_1_integrin^low^/CD133^−^) on the basis of the protocol published previously by Richardson et al [79].

### Irradiation of cells

Cells were irradiated using a RS2000 X-Ray Biological Irradiator, containing a Comet MXR-165 X-Ray Source (Rad-Source Technologies Inc., Suwanee, GA, USA). A dose of 2, 5, 10 and 60 Gy was administered with a dose rate of 0.02 or 0.08 Gy s^−1^.

### Immunofluorescence

Immunocytochemistry was performed as previously described [8]. Antibodies used are listed in Supplementary Table S1.

Images were captured using a Nikon Eclipse TE300 fluorescent microscope (Nikon, Surrey, UK) and were analyzed using Volocity software (Improvision, Perkin Elmer, Waltham, MA, USA). Pseudo-coloring and picture overlay was performed with Velocity software.

### Quantitative real-time PCR for mRNA

Total RNA was extracted from cells using Qiagen RNease mini Kit (Qiagen, Manchester, UK) according to the manufacturer's protocol. RNA was reverse transcribed, using random hexamers (Life Technologies Ltd, Paisley, UK) and reverse transcriptase kit SuperScript III (Life Technologies Ltd, Paisley, UK).

qRT-PCR was conducted using TaqMan gene expression assays (Life Technologies Ltd, Paisley, UK) and the iTaq™ Universal Supermixes (Bio-Rad Laboratories Ltd, Hertfordshire, UK), according to the manufacturer's protocol. Total RNA was extracted from cells using mirVana™ miRNA Isolation Kit (Life Technologies Ltd, Paisley, UK), according to the manufacturer's protocol. miRNA was reverse transcribed, using miScript II RT Kit (Qiagen, Manchester, UK). qRT-PCR for miRNAs was conducted using human specific miScript Primer Assays (Qiagen, Manchester, UK) and the miScript SYBR® Green PCR Kit (Qiagen, Manchester, UK), according to the manufacturer's protocol. All reactions were carried out in triplicate on FrameStar® 96, fully-skirted plate with black frame and white wells for qRT-PCR (4titude Limited, Surrey, UK) in an CFX96 Touch™ Real-Time PCR Detection System (Bio-Rad Laboratories Ltd, Hertfordshire, UK). Expression values are presented relative to the endogenous control gene, RPLP0 for mRNA and U6 small nuclear 6 for miRNA.

### esiRNA and miRNA inhibitor transfection

The miRNA inhibitors anti-hsa-miR-99a-5p miScript miRNA Inhibitor (miR99a-i), Anti-hsa-miR-100-5p miScript miRNA Inhibitor (miR100-i) and the endo-ribonuclease prepared siRNA (esiRNA) esiPARP1, esiSMARCA5, esiSMARCD1 (Sigma-Aldrich Company Ltd, Gillingham, UK) were transfected with Lipofectamine® RNAiMAX Transfection Reagent (Life Technologies Ltd, Paisley, UK) according to the manufacturer's protocol.

### Western blot

After the required treatments, cells were washed with PBS, followed by lysis in RIPA buffer, and the sample buffer for SDS–PAGE was added. The protein concentrations were determined using the Pierce™ BCA Protein Assay Kit (Life Technologies Ltd, Paisley, UK). 20 μg protein per lane were separated by 10% SDS–PAGE and transferred to polyvinylidene difluoride membranes (Millipore, Billerica, MA, USA). Antibodies used were as follows listed in Supplementary Table S1.

Membranes were developed in a GeneGnome XRQ imaging system (Syngene, Cambridge, UK) with BM Chemiluminescence Western Blotting Substrate (POD) (Roche, Welwyn Garden City, UK).

### Clonogenic recovery

Unsorted or CB cells isolated from primary prostate cultures were treated with either 10 nM Dexamethasone (Sigma-Aldrich Company Ltd, Gillingham, UK) dissolved in Ethanol, 1 μM Mifepristone (RU486) (Sigma-Aldrich Company Ltd, Gillingham, UK) dissolved in DMSO for 3 days, and/or irradiated with 5 Gy. According to the different clonogenic potential of the different populations, 100 unsorted or 300 CB cells were plated on to 35-mm collagen I-coated plates (BD Biocoat; BD biosciences) in the presence of irradiated mouse embryonic fibroblast cell line STO cells as feeder cells. Colonies were subsequently scored if they contained more than 32 cells (5 population doublings) usually between 14 to 28 days after treatment [80].

### Flow cytometry

Cultured cells were trypsinised, resuspended in MACs buffer and incubated with antibodies to the Integrin α2 (MCA743PET AbD Serotec, Kidlington, UK) and integrin α6 CD49f (11–0495–80, eBioscience, San Diego, USA) for 20 min at 4°C. Cells were then analyzed on a CyAn-ADP flow cytometer (Beckman Coulter, High Wycombe, UK) and data processed using Summit v4.3 software (Beckman Coulter).

### Live cell count

Collected cells were stained with Trypan Blue (Sigma-Aldrich Company Ltd, Gillingham, UK) and counted using a Neubauer's haemocytometer.

### Cell migration assay

Cells were plated in a 10-cm dish for 48 hours. A wound was created using a 1-ml pipette tip. The width of the wound at 0 and 24 hours was measured using Volocity software (Perkin Elmer, Waltham, MA, USA). The average (of 10 random points) was taken and the relative percentage wound closure at 24 hours with respect to the starting wound size was calculated.

### Statistical analyses

GraphPad Prism 5 (GraphPad Software, La Jolla, CA, USA) was used for statistical analyses. Mann–Whitney U or the Student's t-test was used to determine if two sets of data were significantly different from each other. Correlation analysis was performed by the Pearson's method. Data are presented as mean±standard deviation (SD) unless otherwise specified. All experiments were performed in at least 3 independent replications.

## SUPPLEMENTARY MATERIALS FIGURES AND TABLES


