# Supplementary material for: Inhibition of the glucocorticoid receptor results in an enhanced miR-99a/100-mediated radiation response in stem-like cells from human prostate cancers
Source: Oncotarget. 2016 Jun 21;7(32):51965–80. doi: 10.18632/oncotarget.10207 (PMC5239528; doi:10.18632/oncotarget.10207)
Supplement: Supplementary file 1 [file oncotarget-07-51965-s001.pdf]

Inhibition of the glucocorticoid receptor results in an enhanced miR-99a/100-mediated radiation response in stem-like cells from human prostate cancers

SUPPLEMENTARY FIGURES AND TABLE

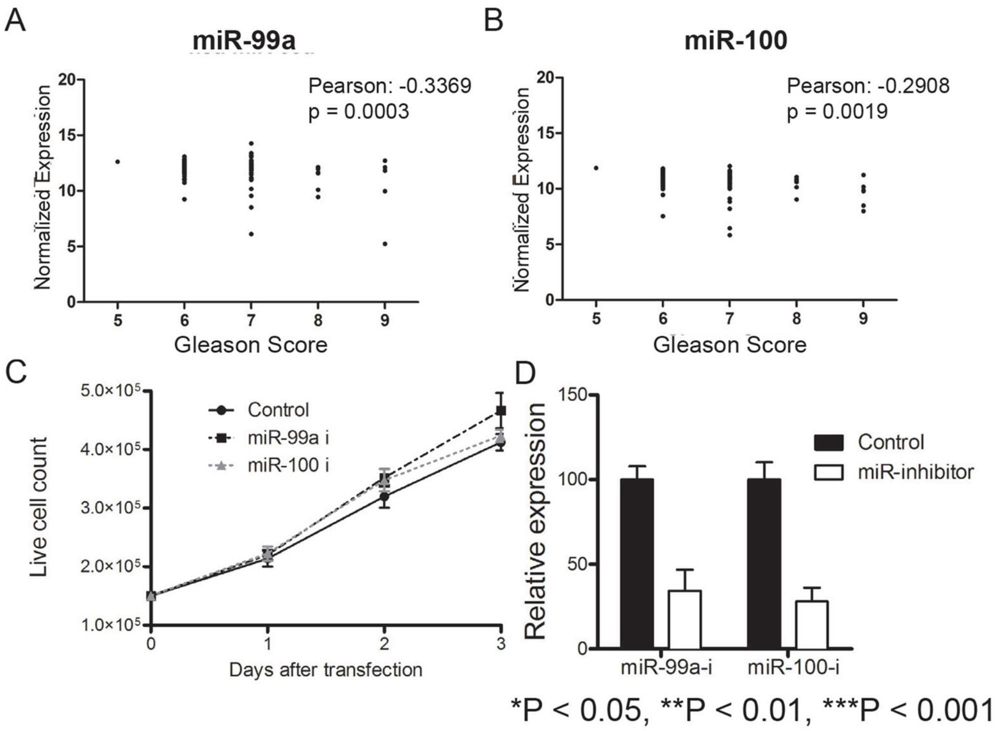

Supplementary Figure S1: A+B. Pearson correlation analysis of miR-99a (A) and miR-100 (B) expression with Gleason Score in the GSE21036 cohort. **C.** Proliferation analysis of malignant CB cells measured by live cell count after miR-99a and miR-100 inhibition (n=3 PCa). **D.** qRT-PCR analysis of miR-99a and miR-100 expression in CB cells 72 hours after mi-R99a and miR-100 inhibitor transfection (n=3 PCa). Data are expressed as mean ± s.d. *P < 0.05, **P < 0.01, ***P < 0.001 (Student's ttest).

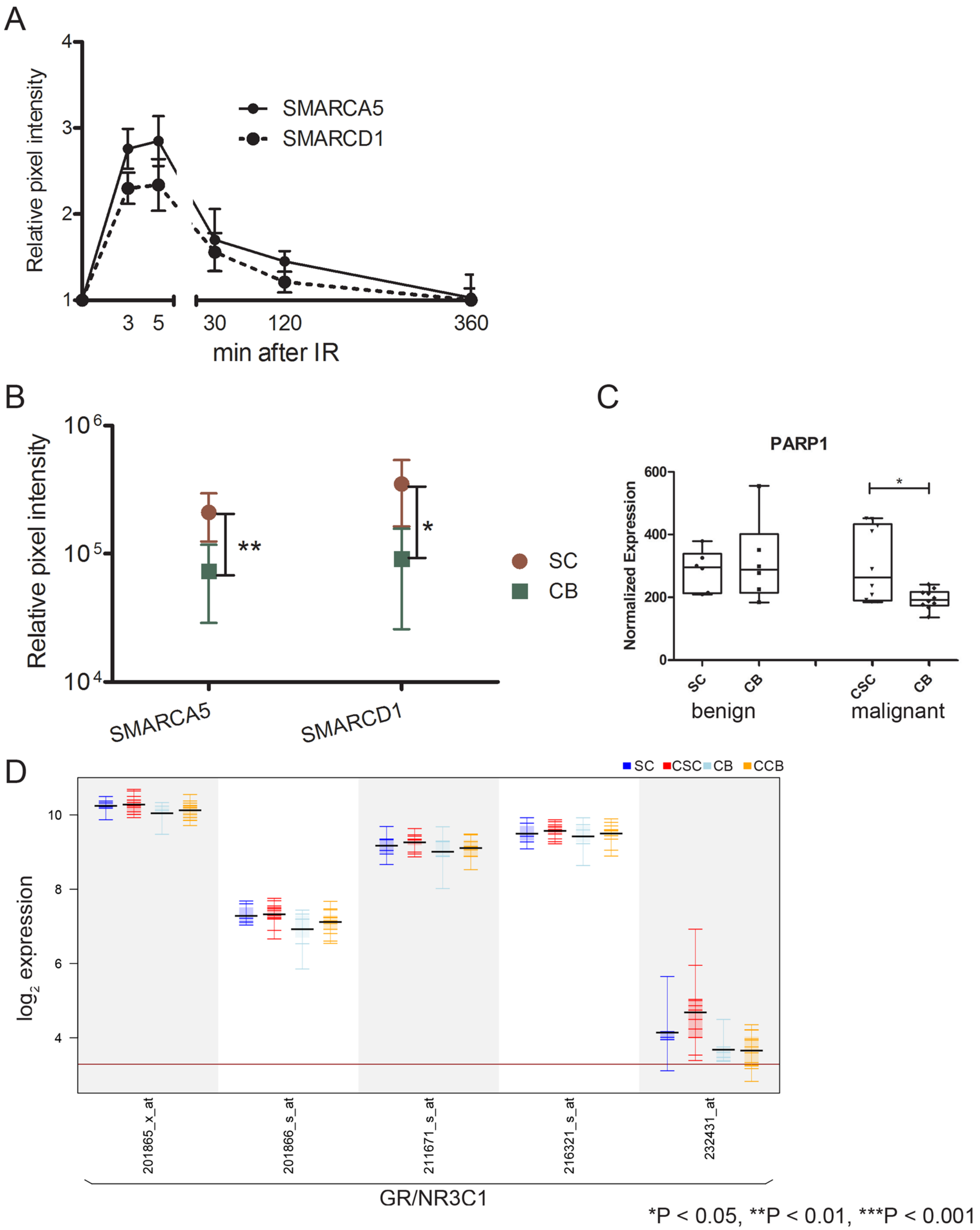

Supplementary Figure S2: A. Rapid recruitment kinetics of SMARCA5 and SMARCD1 to the cell nucleus in SC post 5Gy irradiation. **B.** Quantification of nuclear SMARCA5 and SMARCD1 in SC and CB. Immunofluorescence staining was performed 5 minutes after exposure to 5-Gy radiation (n=3 BPH and PCa, each sample in triplicate). >250 cells/sample were counted. **C.** Normalized microarray expression of PARP1 in SC (s) and CB (c) cells from Birnie et al., 2008 dataset. **D.** Log2 microarray expression of glucocorticoid receptor (GR/NR3C1) probes in benign SC malignant SC (CSC), benign CB, and malignant CB (CBC) from Birnie et al., 2008 dataset. Data are expressed as mean ± s.d. *P < 0.05, **P < 0.01, ***P < 0.001 (Student's ttest).

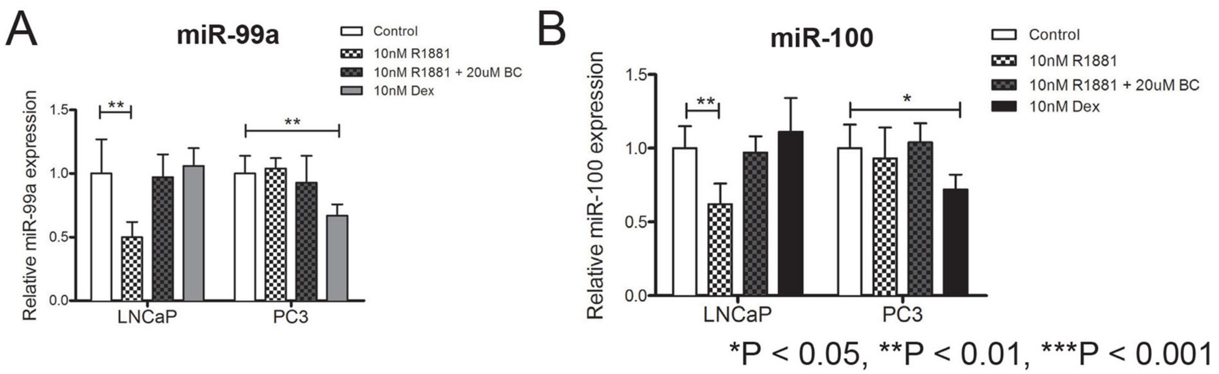

Supplementary Figure S3: A+B. qRT-PCR analysis of miR-99a (A) and miR-100 (B) expression in LNCaP and PC3 cells 72 hours after treatment with R1881, Bicalutamide or Dexamethasone. (n=3). Data are expressed as mean ± s.d. *P < 0.05, **P < 0.01, ***P < 0.001 (Student's ttest).

Supplementary Table S1: Antibody dilutions used for immunofluorescence (IF) and western blot (WB)

		Western Blot	Immunofluorescense
**Name**	**Company**	**Working Dillution**	
Anti-gamma H2A.X (phospho S139) antibody (ab11174)	Abcam, Cambridge, UK		1:200
Anti-SMARCA5 antibody (ab3749)	Abcam, Cambridge, UK	1:500	1:500
Anti-SMARCD1 antibody (611728)	BD Biosciences, San Jose, USA	1:1000	1:100
Phospho-p53 (Ser20) Antibody (#9287)	Cell Signaling Technology, Danvers, USA		1:100
Cleaved Caspase-3 (Asp175) Antibody (#9661)	Cell Signaling Technology, Danvers, USA		1:400
Cleaved PARP (Asp214) Antibody (Human Specific) (#9541)	Cell Signaling Technology, Danvers, USA		1:100
CDH1/E-Cadherin Clone NCH-38 (M3612)	Dako UK Ltd, Cambridge, UK	1:1000	
GAPDH Antibody Mouse monoclonal Clone: 1E6D9 (60004-1-Ig)	Proteintech Europe, Manchester, UK	1:50000	
BRCA1 Antibody (C-20) (sc-642)	Santa Cruz Biotechnology, Inc., Heidelberg, Germany	1:500	
Vimentin Antibody (V9) (sc-6260)	Santa Cruz Biotechnology, Inc., Heidelberg, Germany	1:500	
goat anti-mouse AlexaFluor-488	Life Technologies Ltd, Paisley, UK		1:500
goat anti-rabbit AlexaFluor-568	Life Technologies Ltd, Paisley, UK		1:500
